# Cross-species amplification of 41 microsatellites in European cyprinids: A tool for evolutionary, population genetics and hybridization studies

**DOI:** 10.1186/1756-0500-3-135

**Published:** 2010-05-17

**Authors:** Vincent Dubut, Melthide Sinama, Jean-François Martin, Emese Meglécz, Juliette Fernandez, Rémi Chappaz, André Gilles, Caroline Costedoat

**Affiliations:** 1 Aix-Marseille Université, CNRS, IRD, UMR 6116 - IMEP, Equipe Evolution Génome Environnement, Centre Saint-Charles, Case 36, 3 Place Victor Hugo, 13331 Marseille Cedex 3, France; 2 Montpellier SupAgro, INRA, CIRAD, IRD, Centre de Biologie pour la Gestion des Populations, Campus International de Baillarguet, CS30016, 34988 Montferrier-sur-Lez, France

## Abstract

**Background:**

Cyprinids display the most abundant and widespread species among the European freshwater Teleostei and are known to hybridize quite commonly. Nevertheless, a limited number of markers for conducting comparative differentiation, evolutionary and hybridization dynamics studies are available to date.

**Findings:**

Five multiplex PCR sets were optimized in order to assay 41 cyprinid-specific polymorphic microsatellite loci (including 10 novel loci isolated from *Chondrostoma nasus nasus, Chondrostoma toxostoma toxostoma *and *Leuciscus leuciscus*) for 503 individuals (440 purebred specimens and 63 hybrids) from 15 European cyprinid species. The level of genetic diversity was assessed in *Alburnus alburnus, Alburnoides bipunctatus, C. genei, C. n. nasus, C. soetta, C. t. toxostoma, L. idus, L. leuciscus, Pachychilon pictum, Rutilus rutilus, Squalius cephalus *and *Telestes souffia*. The applicability of the markers was also tested on *Abramis brama, Blicca bjoerkna *and *Scardinius erythrophtalmus *specimens. Overall, between 24 and 37 of these markers revealed polymorphic for the investigated species and 23 markers amplified for all the 15 European cyprinid species.

**Conclusions:**

The developed set of markers demonstrated its performance in discriminating European cyprinid species. Furthermore, it allowed detecting and characterizing hybrid individuals. These microsatellites will therefore be useful to perform comparative evolutionary and population genetics studies dealing with European cyprinids, what is of particular interest in conservation issues and constitutes a tool of choice to conduct hybridization studies.

## Findings

The Cyprinidae family is of special interest for conducting comparative differentiation, evolutionary and hybridization dynamics studies: (i) Cyprinidae is the most abundant and widespread freshwater fish family across the world [1]; and (ii) the Cyprinidae family is characterized by high level of inter-species hybridization (reviewed in [2]). An indirect way to develop microsatellite markers in species with non-sequenced genomes holds in the cross-species amplification of loci previously developed in related species (e.g. [3]). Here, we examined cross-species amplification success of 41 cyprinid-specific polymorphic microsatellite markers, including 10 novel loci.

This was done for 15 European cyprinid species and hybrids (Table 1). They represent 11 geographically widespread European cyprinid species (*Alburnus alburnus, Alburnoides bipunctatus, C. n. nasus, L. idus, L. leuciscus, Rutilus rutilus, Squalius cephalus, Telestes souffia, Abramis brama, Blicca bjoerkna *and *Scardinius erythrophtalmus*) and four endemic species (*C. genei, C. soetta, C. t. toxostoma *and *Pachychilon pictum*). The species sampling represents 12 of the 24 European Cyprinidae genera. We also studied two sets of hybrid specimens. The first one (Table 1; Additional file 1) consists in 48 *Chondrostoma *hybrids specimens (i.e. hybrids between *C. t. toxostoma *and *C. n. nasus*) from the Durance River previously characterized using the mitochondrial cytochrome *b *gene and four nuclear intron sequences [2]. These hybrids (Hy) range from 10% of *C. n. nasus *alleles in their genome (Hy1) to 80% of *C. n. nasus *alleles in their genome (Hy8) (*sensu *[2]). The second set of hybrids consists of 15 individuals (Table 1; Additional file 2) which exhibited an intermediate morphology between two cyprinid species or for which the species identification was not coherent among the different markers (meristic, mitochondrial or microsatellites). All specimens were beforehand identified at the species level using a morphological analysis (we used identification key based on meristic characters [4]) and by sequencing the 5' part of the cytochrome *b *gene (as described in [5]). The results from mitochondrial sequences of all the 440 purebred specimens were congruent with their morphological identification and species assignation could be done without any ambiguity (data not shown).

**Table 1:** European cyprinid species and hybrids: Location and sample size.

Cyprinid species	Hydrographic location	Sample size	
*A. bipunctatus*	Durance River (Southeastern France)	30	
*A. alburnus*	Durance River (Southeastern France)	29	
	Serre-Ponçon Lake (Southeastern France)	12	
*C. genei*	Po River (Northern Italy)	22	
*C. n. nasus*	Allier River (Central France)	32	
	Rhone River (Eastern France)	13	
*C. soetta*	Po River (Northern Italy)	24	
*C. t. toxostoma*	Suran River (Eastern France)	31	
	Serre-Ponçon Lake (Southeastern France)	41	
*L. idus*	Rhine River (Germany)	32	
*L. leuciscus*	Ain River (Eastern France)	29	
*P. pictum*	Orbieu River (Southern France)	28	
*R. rutilus*	Serre-Ponçon Lake (Southeastern France)	37	
*S. cephalus*	Durance River (Southeastern France)	31	
	Serre-Ponçon Lake (Southeastern France)	19	
*T. souffia*	Buech River (Southeastern France)	32	
*A. brama*	Durance River (Southeastern France)	2	
*B. bjoerkna*	Durance River (Southeastern France)	4	
*S. erythrophtalmus*	Durance River (Southeastern France)	2	

***Chondrostoma *hybrids**	**Hydrographic location**	**Sample size**	**Hybrid dilution**

*C. t. toxostoma *× *C. n. nasus *hybrids	Durance River (Southeastern France)	48	Hy1 to Hy8 (see Additional file 1)

**Other cyprinid hybrids' ID **(n = 15)	**Hydrographic location**	**Morphology**	**Mitochondrial DNA**

8Sur1003	Suran River (Eastern France)	*C. t. toxostoma*	*T. souffia*
7SP28	Serre-Ponçon Lake (Southeastern France)	*R. rutilus*	*C. t. toxostoma*
7SP29	Serre-Ponçon Lake (Southeastern France)	*C. t. toxostoma*	*R. rutilus*
7SP31	Serre-Ponçon Lake (Southeastern France)	*C. t. toxostoma*	*C. t. toxostoma*
Gseph29	Serre-Ponçon Lake (Southeastern France)	*R. rutilus */*S. cephalus*	*R. rutilus*
Hsepha	Serre-Ponçon Lake (Southeastern France)	*R. rutilus */*S. cephalus*	*R. rutilus*
Hsephe	Serre-Ponçon Lake (Southeastern France)	*A. alburnus */*R. rutilus*	*A. alburnus*
9AVIBrem1	Durance River (Southeastern France)	*B. bjoerkna*	*R. rutilus*
9AVIBrem2	Durance River (Southeastern France)	*B. bjoerkna*	*R. rutilus*
9AVICheH1	Durance River (Southeastern France)	*A. alburnus */*S. cephalus*	*S. cephalus*
9AVIHyb01	Durance River (Southeastern France)	*R. rutilus */*S. erythrophtalmus*	*S. erythrophtalmus*
9AVIHyb02	Durance River (Southeastern France)	*A. alburnus */*S. cephalus*	*A. alburnus*
9AVIHyb03	Durance River (Southeastern France)	*A. alburnus */*S. cephalus*	*S. cephalus*
Scar26	Po River (Northern Italy)	*C. soetta*	*T. muticellus*
Scar32	Po River (Northern Italy)	*C. soetta*	*T. muticellus*

Ten novel loci were isolated from *Chondrostoma toxostoma toxostoma, Chondrostoma nasus nasus *and *Leuciscus leuciscus *following a protocol detailed elsewhere [6,7]. The program MICROFAMILY[8] was used to discard redundancies by detecting flanking region similarities among different loci. Sixty-nine primers pairs were designed and a total of 10 novel primer pairs were retained (Additional file 3). They were associated with clear amplification pattern, with unambiguous genotype profiles, and were polymorphic for at least one of the 15 cyprinid species. Thirty-one primers pairs from previously described microsatellite loci [6,7,9-19] were then integrated into the protocol. These loci were combined into multiplex PCR kits, along with the 10 novel loci. Overall, a total of 41 loci were combined into five multiplex PCR kits (Additional file 3). Amplifications and genotyping were conducted using reagents and protocols described previously [6].

The size range of the applicable markers with a common fluorescent dye did not overlap (Additional file 3), except for CtoG-075 and Lid8 in *A. alburnus *and BL1-T2 and CypG24 in *L. idus*. Nevertheless, the differences in genotype profiles allowed to unambiguously discriminating alleles among loci. The mean rate of amplification success per species is 95.1% (i.e. 39/41 loci) and the mean rate of polymorphic loci per species (excluding *A. brama, B bjoerkna *and *S. erythrophtalmus *because of too small sample size) is 76.6% (i.e. about 31/41 loci) (Table 2). Moreover, 23 loci (namely BL1-2b, BL1-30, BL1-84, BL1-98, BL1-153, Ca1, CtoA-247, CtoF-172, CtoG-216, CypG24, IV04, LceC1, LleA-071, LleC-090, Lsou05, Lsou08, Lsou19, Lsou34, N7K4, Ppro132, Rru4, Rser10 and Z21908) displayed amplification success in all the species tested here. The vast majority of cross-species amplifications from the European cyprinid species resulted in PCR fragments with proper microsatellite chromatograms and expected allele sizes, indicating homologous loci. The 41 retained sequences containing microsatellites were BLASTed against the complete genome of *Danio rerio *(e-value = 1E-20) as a proxy for testing loci homology. This procedure makes the assumption that chromosome topology is well conserved across cyprinid species (e.g. [20]). Eighteen of the 41 sequences produced a significant hit and were mapped onto the *D. rerio *genome without ambiguity (Table 3).

**Table 2:** Matrix of the number of polymorphic and amplified loci shared between species.

	***Ab***	***Aa***	***Cg***	***Cn***	***Ct***	***Cs***	***Li***	***Ll***	***Pp***	***Rr***	***Sc***	***Ts***	Abr*	Bb**	Se*
*A. bipunctatus*	**31/35**	28	26	29	23	26	25	23	19	24	24	26	12	22	9
*A. alburnus*	34	**34/37**	31	32	26	26	28	27	21	27	28	30	14	24	10
*C. genei*	33	36	**35/40**	34	28	26	31	26	22	27	29	31	15	24	11
*C. n. nasus*	34	37	40	**37/41**	29	27	31	27	22	28	29	33	15	26	12
*C. t. toxostoma*	34	37	40	41	**31/41**	24	29	25	18	26	25	30	14	22	10
*C. soetta*	34	37	39	40	40	**28/40**	25	24	16	25	25	26	13	20	8
*L. idus*	32	35	39	39	39	38	**32/39**	26	19	28	25	29	13	23	10
*L. leuciscus*	34	36	38	39	39	38	37	**29/39**	18	26	24	26	14	22	9
*P. pictum*	31	34	37	38	38	37	36	36	**24/38**	19	22	20	11	19	8
*R. rutilus*	34	37	39	40	40	39	38	39	37	**30/40**	25	27	14	23	10
*S. cephalus*	32	36	37	38	38	37	36	37	35	38	**32/38**	27	15	22	10
*T. souffia*	33	36	39	40	40	39	38	38	37	39	37	**33/40**	15	23	11
A. brama*	34	37	39	40	40	39	38	39	37	40	38	39	**16/40**	13	6
B. bjoerkna**	32	35	37	38	38	37	36	37	37	39	36	37	38	**27/38**	10
S. erythrophtalmus*	33	35	37	38	38	37	36	37	36	38	36	37	38	36	**12/38**

In upper diagonal, shared polymorphic loci; in lower diagonal, shared amplified loci between species; in diagonal, polymorphic loci/amplified loci for each species. Loci for which homozygotes with null alleles were detected and those exhibiting potential paralogous (see Additional file 3) were discarded. Only two (*) and four (**) individuals genotyped: number of polymorphic loci most likely underestimated.

**Table 3:** Position of the 18 microsatellite loci that match with *Danio rerio *(*Dr*) genome.

*Dr *chromosome	Numbers of sequences hitting *Dr *chromosomes	Position (in Mb) on Chromosome (locus)				Chromosome length (in Mb)
Dr01	2	50 (CnaD-112)	53 (CnaB-030)			56
Dr02	1	44 (BL1-30)				54
Dr05	4	11 (Ca1)	32 (BL1-84)	46 (Ca3)	56 (Lid8)	70
Dr06	1	44 (Lsou29)				59
Dr07	2	42 (CypG24)	45 (CtoA-256)			70
Dr08	2	27 (LleC-090)	35 (CnaF-177)			56
Dr12	1	28 (CtoA-247)				47
Dr13	1	57 (CtoG-216)				53
Dr17	1	28 (BL2-114)				52
Dr23	1	40 (LCO3)				46
Dr24	2	33.8 (BL1-98)	34 (Z21908)			40

For populations with more than 20 samples, GENEPOP 4.0 [21] was used to: (i) test for the Hardy-Weinberg (HW) equilibrium, (ii) estimate the heterozygosity for all loci and populations, and (iii) test linkage disequilibrium (LD) among loci within populations. MICRO-CHECKER ver. 2.2.3 [22] was used to analyze the causes of departures from HW equilibrium: real HW disequilibrium, null alleles or scoring errors (often resulting from stuttering). Among the 41 loci, we noticed five cases where homozygotes with null alleles were detected (Additional file 1): Lsou29 in *A. bipunctatus *and *A. alburnus*; CtoE-249 in *A. alburnus*; BL1-T2 in *L. leuciscus*; and CnaD-112 and LCO5 in *B. bjoerkna*. These cases were discarded from the analyses dealing with HW and LD analyses. Most of the 41 loci were at HW equilibrium after False Discovery Rate (FDR) correction (modified [23] and applied within each species). Considering all loci and species together, we found 1218 cases of polymorphic pattern among which only sixteen cases (<1%) displaying HW disequilibrium (see Additional file 4). Most loci with HW disequilibrium showed significant excess of homozygotes. It is noticeable that all *A. bipunctatus *individuals exhibited two alleles at locus LCO3. Regarding their size, these alleles are very close to the variation range observed in the other cyprinid species, suggesting that this locus has experimented duplication within the genome of *A. bipunctatus*. This locus was therefore discarded from the *A. bipunctatus *data for further statistical analyses. The MICRO-CHECKER analyses indicated that homozygote excess at loci with HW equilibrium departures was mainly caused by the presence of null alleles. A total of 6152 pairwise comparisons were submitted to LD analyses, and LD was tested with 515 pairwise comparisons per species in average. After FDR correction, only 36 pairs of loci exhibited LD (0.006% of the total pairwise comparisons). A noticeable higher number of pairs of loci at LD were found in *L. idus *(21/496 of pairwise comparisons) and *C. n. nasus *(Allier River) (7/666) (Additional file 4). Interestingly, LD was found between BL1-98 and Z21908 in two cases (in *C. n. nasus *and *L. idus*), in agreement with their relative vicinity (253 Kb) on the *D. rerio*'s chromosome 24 (Table 3). Additionally, three other pairs of loci displayed LD in two or more species: LleA-150 and Lsou34 in *A. bipunctatus *and *L. idus*; BL1-T2 and Z21908 in *C. n. nasus *and *L. idus*; and BL1-2b and BL1-T2 in *C. genei, C. n. nasus, C. t. toxostoma *from Serre-Ponçon Lake and *L. idus *(Additional file 4).

A Bayesian-based approach was used to search for the occurrence of independent genetic groups (K) in the microsatellites dataset (STRUCTURE 2.2 [24]; http://pritch.bsd.uchicago.edu). The burn-in length was set to 100,000 followed by 1,000,000 iterations within a Markov Chain Monte Carlo (MCMC). The 'admixture model' and the 'I-model' (independent allele frequencies) were used, with no prior population information. Parameter K was chosen to vary from 1 to 20 and five repeats were run for each K. We selected the K value for which the posterior probability of the data, Ln P(D), was maximized. Each individual was assigned to the inferred clusters according to the results from the simulation procedures (parameter "Q" representing the estimated membership coefficients for each individual in each cluster). Because choosing K can be difficult *a priori *(although in our case the number of species is known), we combined two main approaches [25]: i) choosing K that maximizes the posterior probability of the data Ln P(D); ii) using the formula [Ln P(D)_k _- Ln P(D)_k-1_], where Ln P(D) is the estimated posterior probability of the data conditional to K. *A. brama, B. bjoerkna *and *S. erythrophtalmus *individuals were discarded from these analyses due to their too limited sample size (see [26]). Moreover, the pattern of allelic frequency differentiation between species was explored through Factorial Correspondence Analyses (FCA) using GENETIX ver. 4.05.2 [27]. In addition, for the *C. n. nasus *and *C. t. toxostoma *specimens and their hybrids, the Bayesian clustering method implemented in the program NEWHYBRID[28] was used to assign individuals to different genotypic classes (parental, F1, F2 or backcrosses). The method computes, by MCMC method, the Bayesian posterior probability that an individual in a sample belongs to different hybrid classes (F1, F2, and backcrosses) while simultaneously estimating allelic frequencies for parental species. The program was run five times with varying lengths of burn-in period and numbers of sweeps, as recommended by the authors.

**Figure 1:**
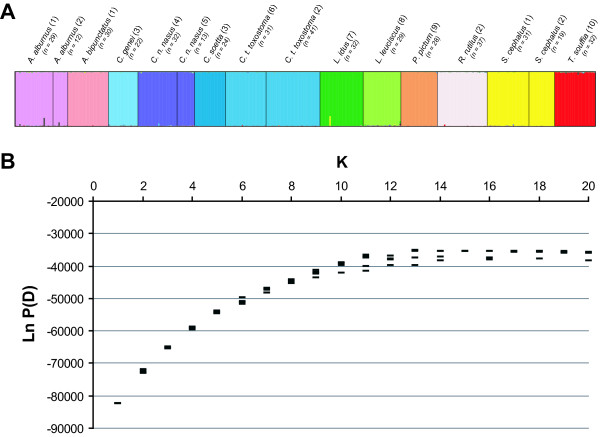
**Graph of the STRUCTURE run applied to 12 cyprinid species with K = 13 (A) and Ln P(D) values distribution for K = 2-20 (B) for 41 microsatellites**. Numbers between parentheses refer to the sample location: 1, Durance River (southeastern France); 2, Serre-Ponçon Lake (southeastern France); 3, Po River (northern Italy); 4, Allier River (central France); 5, Rhone River (southeastern France); 6, Suran River (eastern France); 7, Rhine basin (Germany); 8, Ain River (eastern France); 9, Orbieu River (southern France); 10, Buech River (southeastern France).

Based on either all the 41 or 23 microsatellites loci, K = 13 was obtained from the Ln P(D) analyses for K parameter determination (see Figure 1), which approximate well the number of analysed cyprinid species. Within species, correct assignment score ranged from 97% to 99% both with 23 loci and 41 loci. Additionally, FCA could separate the 15 cyprinid analysed species (axes 1 and 2; Additional files 5 and 6). It is worth noting that the graphical discrimination was increased when the two outliers (*A. bipunctatus *and *P. pictum*) were discarded. Moreover, as highlighted by the results from the different populations of *A. alburnus, C. n. nasus, C. t. toxosmtoma *or *S. cephalus*, the quality of species identification, both in FCA or STRUCTURE analyses, did not depend on the population sampled (Figure 1; Additional files 5 and 6).

**Figure 2:**
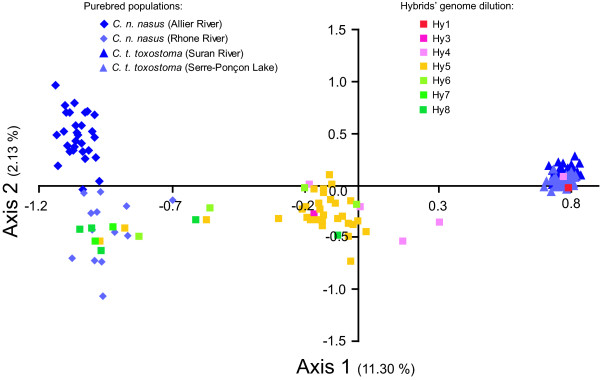
**Biplot representation of the 41 microsatellites-based FCA conducted on *Chondrostoma *specimens**. Were included in the analysis *C. n. nasus *specimens from Allier (n = 32) and Rhone (n = 13) Rivers, *C. t. toxostoma *specimens from Suran River (n = 31) and Serre-Ponçon Lake (n = 41) and their hybrids (n = 48) from Durance River (France).

The genotypic distribution of the *Chondrostoma *hybrids specimens compared to their parental species (*C. t. toxostoma *and *C. n. nasus*) is summarized in Figure 2. The two purebred *C. t. toxostoma *populations can hardly be differentiated on axes 1 and 2 of the FCA, whereas a differentiation between the *C. n. nasus *sampled in Allier River and those sampled in Rhone River is displayed on axis 2. A strong coherence was found between the genome dilution as defined by [2] and the distribution of the hybrids between the two parental species. Indeed, Hy1 genotypes were assigned toward the *C. t. toxostoma *species whereas Hy8 were assigned toward the *C. n. nasus *species. F1 hybrids, which correspond to Hy4 and Hy5 depending on their mitochondrial sequence, were mainly intermediate between the two parental species. However, these F1 hybrids are not strictly homogeneous and the assignment scores fluctuate (Additional file 1; see also Figure 2). Variations found at the level of assignment scores for Hy4 and Hy5 hybrids may be caused by the limited number of markers (n = 5) used by [2], although they were discriminative markers. As for the second hybrid group, thirteen different hybrid combinations have been identified. Most of the hybrids revealed being introgressed individuals (i.e. individuals assigned to one species based on mitochondrial cytochrome *b *gene or morphology and to another species based on microsatellites), although most of them exhibited an intermediate morphology (Additional file 2).

To our knowledge, the development of large number (n > 20) of polymorphic microsatellite markers applicable to European cyprinid species has never been achieved to date (but see for instance [3]). Moreover, through the species sampling designed in this study, we demonstrated their applicability within half of the European cyprinid genera, with 24 to 37 polymorphic loci per species. A common set of 23 markers enabled the comparison of 15 species and hence allows genetic variability and recombination to be compared directly for these species. Furthermore, normalization of the PCR conditions and multiplexing make faster and cost effective the genotyping of the 41 loci. The high number of loci and wide applicability to the European cyprinid species make the developed set of markers a powerful tool for: (i) studies dealing with genetic diversity and structure of the European cyprinid species (these markers will notably be useful to assess the impact of anthropogenic factors on the cyprinid genetic diversity, and will be useful for conservation and environmental monitoring purposes); (ii) comparative studies dealing with the evolutionary pattern and history of a large set of species; (iii) species identification; and (iv) developing knowledge in hybridization processes and dynamics in Cyprinids. More specifically, the analysis of a large number of genetic markers (see [29]) will significantly improve the understanding of the relative impact of inter-species interactions, response to environmental effects and ecological trade-offs in cyprinid hybrid zones (as initiated by [30]).

## Competing interests

The authors declare that they have no competing interests.

## Authors' contributions

MS, VD, JFM and JF carried out the molecular genetics laboratory work. VD, MS, EM and CC performed the statistical analyses. VD, CC and MS drafted the manuscript. AG, VD and CC conceived the study. RC participated to the financial support. All authors read, contributed to and approved the final manuscript.

## Supplementary Material

Additional file 1 **STRUCTURE (K = 2) and NEWHYBRID assignment scores for the 48 *Chondrostoma *hybrids using 41 microsatellites**. Excel Table containing STRUCTURE (K = 2) and NEWHYBRID assignment scores for the 48 *Chondrostoma *hybrids using 41 microsatellites. Click here for file

Additional file 2 **Characterization and STRUCTURE (K = 15) assignment scores for the 15 cyprinid hybrids using 41 microsatellites**. Excel Table containing the characterization and STRUCTURE (K = 15) assignment scores for the 15 cyprinid hybrids using 41 microsatellites. Click here for file

Additional file 3 **Microsatellite loci, multiplex PCR conditions, levels of variability and amplifiability of 41 microsatellite loci in 15 cyprinid species**. Excel Table describing the microsatellite loci, multiplex PCR conditions, levels of variability and amplifiability of 41 microsatellite loci in 15 cyprinid species. Click here for file

Additional file 4 **Pairs of loci found to be at Linkage Disequilibrium in 15 cyprinid species**. PDF file reporting the pairs of loci found to be at Linkage Disequilibrium in 15 cyprinid species Click here for file

Additional file 5 **Biplot representations of the FCA for 15 cyprinid species (A) or 13 cyprinid species (excluding *A. bipunctatus *and *P. pictum*) (B) using 41 microsatellites**. PDF file containing the biplot representations of the FCA for 15 cyprinid species (A) or 13 cyprinid species (excluding *A. bipunctatus *and *P. pictum*) (B) using 41 microsatellites. Click here for file

Additional file 6 **Biplot representations of the FCA for 15 cyprinid species (A) or 13 cyprinid species (excluding *A. bipunctatus *and *P. pictum*) (B) using 23 microsatellites**. PDF file containing the biplot representations of the FCA for 15 cyprinid species (A) or 13 cyprinid species (excluding *A. bipunctatus *and *P. pictum*) (B) using 23 microsatellites. Click here for file
