# Supplementary material for: Cross-species amplification of 41 microsatellites in European cyprinids: A tool for evolutionary, population genetics and hybridization studies
Source: BMC Res Notes. 2010 May 17;3:135. doi: 10.1186/1756-0500-3-135 (PMC2883988; doi:10.1186/1756-0500-3-135)
Supplement: Additional file 4 — Pairs of loci found to be at Linkage Disequilibrium in 15 cyprinid species. PDF file reporting the pairs of loci found to be at Linkage Disequilibrium in 15 cyprinid species [file 1756-0500-3-135-S4.PDF]

Additional File 4 - Pairs of loci found to be at Linkage Disequilibrium

Species	Locus 1	Locus 2	P-Value	P-value rank	FDR value
A. bipunctatus	LleA-150	Lsou34	0.0000	1	-0.000101
C. genei	BL1-2b	BL1-T2	0.0000	1	-0.000084
C. nasus	BL1-2b	BL1-T2	0.0000	1	-0.000075
	BL1-2b	Z21908	0.0000	4	-0.000300
	BL1-84	LleA-029	0.0000	3	-0.000225
	BL1-98	Z21908	0.0000	5	-0.000375
	BL1-61	CnaD-112	0.0000	2	-0.000150
	BL1-T2	Lsou08	0.0002	7	-0.000376
	BL1-T2	Z21908	0.0000	6	-0.000450
*C. toxostoma**	BL1-2b	BL1-T2	0.0000	1	-0.000062
L. idus	BL1-2b	BL1-30	0.0000	1	-0.000101
	BL1-2b	BL1-T2	0.0000	3	-0.000302
	BL1-2b	Z21908	0.0012	20	-0.000804
	BL1-30	BL1-153	0.0000	2	-0.000201
	BL1-30	LleA-029	0.0000	5	-0.000503
	BL1-30	LleC-090	0.0000	8	-0.000805
	BL1-30	Lsou19	0.0000	10	-0.001006
	BL1-98	BL1-T2	0.0000	4	-0.000402
	BL1-98	Z21908	0.0000	13	-0.001308
	BL1-T2	Z21908	0.0000	14	-0.001408
	CtoF-172	LleC-090	0.0008	19	-0.001137
	CtoF-172	Lsou05	0.0003	18	-0.001521
	CtoF-172	N7K4	0.0002	15	-0.001343
	LceC1	LleA-029	0.0000	6	-0.000604
	LCO1	LleA-150	0.0000	7	-0.000704
	LleA-029	LleC-090	0.0000	9	-0.000905
	LleA-029	Lsou19	0.0003	17	-0.001432
	LleA-150	Lsou34	0.0000	11	-0.001107
	LleC-090	Lsou05	0.0002	16	-0.001396
	LleC-090	N7K4	0.0020	21	-0.000083
	Lsou05	N7K4	0.0000	12	-0.001207
T. souffia	BL1-30	CypG24	0.0001	3	-0.000208
	Ca3	CtoA-256	0.0000	1	-0.000103
	CtoA-256	Lid8	0.0000	2	-0.000205

* from Serre-Ponçon Lake
