# Supplementary material for: Cross-species amplification of 41 microsatellites in European cyprinids: A tool for evolutionary, population genetics and hybridization studies
Source: BMC Res Notes. 2010 May 17;3:135. doi: 10.1186/1756-0500-3-135 (PMC2883988; doi:10.1186/1756-0500-3-135)
Supplement: Additional file 6 — Biplot representations of the FCA for 15 cyprinid species (A) or 13 cyprinid species (excluding A. bipunctatus and P. pictum) (B) using 23 microsatellites. PDF file containing the biplot representations of the FCA for 15 cyprinid species (A) or 13 cyprinid species (excluding A. bipunctatus and P. pictum) (B) using 23 microsatellites. [file 1756-0500-3-135-S6.PDF]

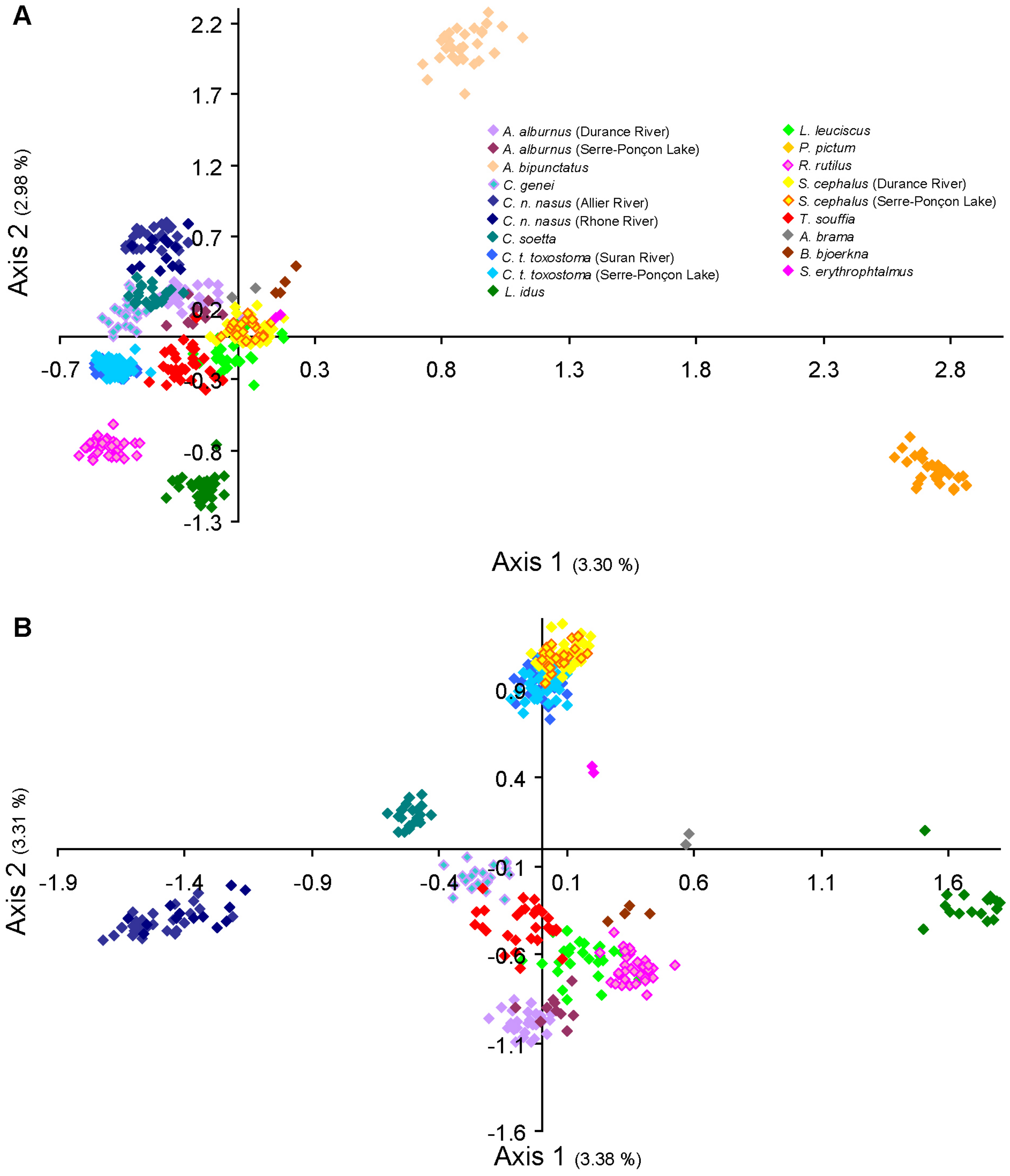

Additional file 6 - Biplot representations of the FCA for 15 cyprinid species (**A**) or 13 cyprinid species (excluding *A. bipunctatus* and *P. pictum*) (**B**) using 23 microsatellites.
